# Sex differences in intrusive memories following trauma

**DOI:** 10.1371/journal.pone.0208575

**Published:** 2018-12-06

**Authors:** Chia-Ming K. Hsu, Birgit Kleim, Emma L. Nicholson, Daniel V. Zuj, Pippa J. Cushing, Kate E. Gray, Latifa Clark, Kim L. Felmingham

**Affiliations:** 1 Division of Psychology, School of Medicine, University of Tasmania, Hobart, Tasmania, Australia; 2 Department of Psychiatry, Psychotherapy and Psychosomatics, University Hospital Zurich, Zurich, Switzerland; 3 Department of Psychology, Swansea University, Singleton Park, Wales, United Kingdom; 4 School of Psychological Sciences, University of Melbourne, Parkville, Victoria, Australia; Harvard Medical School, UNITED STATES

## Abstract

**Background:**

A key mechanism thought to underlie Posttraumatic Stress Disorder (PTSD) is enhanced emotional memory consolidation. Recent evidence in healthy controls revealed that women have greater negative memory consolidation following stress relative to men. This study examined emotional memory consolidation in women and men with PTSD, and in trauma-exposed and non-trauma controls to test the hypothesis that emotionally negative memory consolidation would be greater in women with PTSD.

**Method:**

One hundred and forty-seven men and women (47 with PTSD, 49 trauma-exposed controls, and 51 non-trauma controls) completed an emotional memory task where they looked at negative, neutral and positive images from the International Affective Picture System (IAPS). Delayed recall and an intrusive memory diary were completed two days later.

**Results:**

Women displayed greater recall, and reported more negative intrusive memories than men. A gender x group interaction effect showed that both women with PTSD and trauma-exposed women reported more intrusive memories than women without trauma exposure or men.

**Conclusion:**

This study provided preliminary evidence of sex differences in intrusive memories in those with PTSD as well as those with a history of trauma exposure. Future research should include measures of sex hormones to further examine sex differences on memory consolidation in the context of trauma exposure and PTSD.

## Introduction

Posttraumatic stress disorder (PTSD) is characterised by intrusive memories that are involuntary, distressing and difficult to control [1, 2]. Epidemiological studies reveal that women develop PTSD at higher rates than men, even after controlling for type of trauma exposure [3–6], rate of exposure and history of traumatisation [7]. Less consistent sex differences are found in military samples, however, military populations are subject to selection biases and methodological inconsistencies are common [8]. A meta-analysis found a moderately higher risk of PTSD in women exposed to combat trauma [8] and recently, a large study with more than 34,000 participants revealed a significant increase in PTSD rates in women across a broad range of trauma types, including combat [6].

The mechanisms underlying this higher risk in women remain unclear and are likely to be multifactorial, including social factors such as social and gender roles, cognitive factors, and biological factors. Some researchers have suggested that the sex difference in the prevalence of PTSD is due to the gendered nature of trauma exposure [9], as women are exposed to more sexual and interpersonal violence than men [10], and interpersonal trauma is associated with higher rates of PTSD [11]. However, previous studies have examined risk for PTSD in women and men whilst controlling for exposure to sexual violence and concluded that the increased exposure of females to sexual violence cannot fully account for sex differences [4, 5]. The large epidemiological study noted above found that increased PTSD rates were observed in women across most trauma types (17 out of 19) and that the greater rates of PTSD were not attributable to differences in trauma type, but were likely explained by sex differences in reactivity to the trauma [6]. These latter researchers suggested there may be a particular influence of biological factors, such as stress reactivity and endocrine response, influencing this differential risk for PTSD [6].

A key biological model proposes that PTSD is a result of an over-consolidation of emotional memories following trauma. During the traumatic event, heightened arousal and the release of stress hormones (noradrenaline and cortisol) are thought to lead to an over-consolidation of the trauma memory, resulting in a stronger memory trace that is readily primed by trauma reminders [12]. The subsequent retrieval of the trauma memory accompanied by heightened arousal creates a positive feedback loop that leads to more negative and frequent intrusive memories [13]. Brain imaging and pharmacological studies in PTSD suggested that the reduced activity in medial prefrontal cortex alongside increased amygdala activation and elevated noradrenergic activity might result in enhanced emotional memories [13, 14]. A recent study examined the role of stress hormones during the encoding of emotional images and found, in line with these models, that the interaction of noradrenaline and cortisol during encoding was associated with increased negative intrusive memories in people with PTSD [15].

Empirical evidence in healthy controls suggested that emotional memory consolidation differs in men and women. A study of autobiographical memory revealed that women displayed more accurate and detailed memories of both personal and non-personal experience, especially when the memories involved emotional content, whether positive or negative [16, 17]. Further studies revealed greater memory enhancements in women than men, and this occurs more rapidly and vividly in women [18, 19]. Healthy young women were also found to have greater memory enhancement for neutral stimuli that preceded emotional images compared to men [20].

Many emotional memory studies have employed deliberate recall methods that reflect strategic memory retrieval processes associated with hippocampal activation [21]. When examining memory consolidation in individuals with PTSD or a history of trauma, intrusive memories are also of particular interest and relevance. Intrusive memories are triggered by the environment or occur spontaneously. They are typically sensory-perceptual memories that are vivid [22], fragmented [23] and poorly contextualized into autobiographical memory [24]. The frequency of intrusive memories might be associated with how memories were processed, as healthy individuals reported more intrusions after they attempted to suppress distressing stimuli [25]. Similarly, women with PTSD who attempted to suppress their thoughts experienced a rebound effect in trauma-related thoughts, while the non-PTSD group did not display the same effect [26].

Despite the greater negative memory consolidation and prevalence of PTSD in women, no studies to our knowledge have examined sex differences in intrusive memories among the PTSD population. Accordingly, this study examined recall and intrusive memories of emotional stimuli in women and men with PTSD, in trauma-exposed and non-trauma controls. It was hypothesised that women with PTSD would display particularly greater negative memory recall and negative intrusive memories than men in all groups and women in the control groups.

## Method

### Participants

Participants were 147 individuals (90 females, 57 males) of Caucasian background recruited from university students and community centres. This included a subset of 58 individuals from a previous memory study examining the role of PTSD and stress hormones [15]. An a-priori power analysis for mixed-factorial ANOVA was conducted in G*Power (Version 3.1.9.3) to determine a sufficient sample size using an alpha value of 0.05, a power of 0.95 and a small to moderate effect size (η_p_^2^ = 0.05). The analysis estimated a required total sample size of 120.

DSM-IV-TR criteria were used in this study, as data collection commenced before the DSM-5 tools were available. The experience of trauma was assessed using Traumatic Events Questionnaire (TEQ; [27]) and PTSD was assessed using the PTSD checklist (PCL; [28]), a standardized and well-validated instrument that enables both ordinal symptom severity and diagnosis of PTSD.

Participants were as PTSD if they had experienced a Criterion A trauma and met DSM-IV-TR diagnostic criteria for PTSD (n = 47, 28 females, 19 males, age M = 31.94, SD = 14.55) according to the PCL or if they reported a subclinical status for PTSD (defined as having a total PCL score >40). Trauma-exposed controls (TC) were defined as those who had experienced a criterion A trauma, but did not meet the diagnostic criteria for PTSD (n = 49, 30 females, 19 males, age M = 27.37, SD = 10.32), and non-trauma controls (NC) had never experienced a criterion A trauma (n = 51, 32 females, 19 males, age M = 22.47, SD = 7.38).

The inclusion of the TC group is to control for effects of trauma exposure versus effects of PTSD. This study excluded those who were aged under 18 or over 65 years, pregnant, reported a history of neurological damage or traumatic brain injury, history of psychosis or mania, and heavy substance abuse (Alcohol Use Disorders Identification Test [AUDIT] total score > 15 or were using illicit drugs more regularly than once a week). The research protocol was approved by the Tasmania Social Sciences Human Research Ethics Committee.

### Experimental memory task procedure

Each participant completed the study by attending two sessions separated by a two-day interval. To prevent rehearsal or priming effects, participants were informed that the study examined the impact of arousal on perception of emotional images. They were fully debriefed at the end of the second testing session.

In the first session (encoding session), participants provided informed consent, and completed questionnaires to report their moods and symptoms, including the Depression Anxiety and Stress Scales (DASS; [29]), TEQ and PCL. Participants watched 60 images from the International Affective Picture System (IAPS; [30]) in three blocks according to the pictures’ level of valence (1 = extremely unpleasant, 9 = extremely pleasant) and arousal (1 = very calm, 9 = very aroused) ratings. These included 20 emotionally negative images (mean valence = 2.30, mean arousal = 6.18), 20 neutral images (mean valence = 4.99, mean arousal = 2.75), and 20 positive images (mean valence = 7.49, mean arousal = 4.42). The content of the negative images included assault, violence, accidents and natural disasters. The three blocks of images were shown on a 14-inch coloured computer screen in a randomized counterbalanced order. Images within the same block were shown in random order. Each image was shown for six seconds before the screen automatically switched to the next image slide. Participants were asked to direct their full attention to the images. Participants were informed that they would be asked to complete some similar tasks in the second session after two days.

In the second testing session (recall session), participants were given a surprise free recall task in which they were asked to write down descriptions of as many images as they could recall from the encoding session using a standardized procedure [15, 31]. Two independent raters matched these written descriptions to IAPS images. Descriptions that could not be linked to a specific image were recorded as non-responses. All responses were assessed by two independent raters to determine the accuracy of recall with the percentage of agreement 94%. To assess intrusive memories, participants completed an intrusive memory diary following previously published procedures [15]. The experimenter explained the definition and characteristics of intrusive memories–that is, memories of the images they were shown that have occurred involuntarily and spontaneously in the last two days; and it does not include memories that emerged when they were deliberately thinking about this experiment. Participants were asked to write down a description of any intrusive memories of the IAPS images. All descriptions of intrusive images provided in this study could be linked to a specific image. Participants were also asked, “Did you try to suppress or block any of the negative images?” Lastly, participants were asked to rate the valence and arousal of each image using the scales endorsed by IAPS [30].

### Statistical analyses

Gender distribution across groups was analysed using a 2 (Sex [female, male]) x 3 (Group [PTSD, TC, NC]) Chi-square test of independence. Type of trauma distribution across groups and gender was analysed using separate 2 (Trauma type [interpersonal, non-interpersonal]) x 3 (Group [PTSD, TC, NC]) and 2 (Trauma type Trauma type [interpersonal, non-interpersonal]) x 2 (Sex [female, male]) Chi-square test of independence. Age and symptom data were analysed by separate 2 (Sex [female, male]) x 3 (Group [PTSD, TC, NC]) univariate analyses of variance (ANOVA). The numbers of recalled images, intrusive memories and picture rating data (subjective ratings for valence and arousal) were analysed by separate 2 (Sex [female, male]) x 3 (Group [PTSD, TC, NC]) x 3 (Valence [negative, neutral, positive]) mixed factorial ANOVAs.

All analyses were performed using SPSS (version 21.0, IBM Corp., Armonk, NY 2012). If there was significant sphericity in the data indicating a violation of the statistical assumption in ANOVA, Greenhouse-Geisser corrections were used when epsilon < .75 and Huynh-Feldt corrections were used when epsilon > .75 [32]. Sidak post-hoc pairwise comparisons were used to further assess specific group differences if there were significant main effects or interaction effects. An alpha value of *p* < .05 was used for all analyses.

## Results

### Demographic and clinical data

Table 1 presents the means and standard deviations for clinical data across the groups. In terms of age, there was a group main effect, *F*(2, 141) = 7.90, *p* = .001, such that age was significantly greater in the PTSD group (*M* = 31.94, *SD* = 14.55) than the NC group (*M* = 22.47, *SD* = 7.38). To control for this group difference, analyses were repeated taking age as a covariate (for ANCOVA analyses, see S1 File). There was no significant difference in age for men and women, *F*(1, 141) = 3.44, *p* = .066, and no significant interaction in age between group and sex, *F*(2, 141) = 0.51, *p* = .60. The distribution of males and females across groups was examined using Chi-square analysis, which revealed that the association between sex and group was non-significant, *X*^2^ (2, N = 147) = .10, *p* = .95.

**Table 1:** Mean scores, standard deviations and F-statistics of clinical scales across sex and groups.

Variable		PTSD (*n* = 47)	TC^a^ (*n* = 49)	NC^b^ (*n* = 51)	Effects	Statistics	*p*	η_p_^2^
DASS								
Depression	F	19.07	4.80	5.13	Group	*F* = 39.88	< .001	0.36
		(10.53)	(5.45)	(6.72)	Gender	*F* = 1.65	0.20	0.012
	M	14.95	6.32	2.74	Group x Gender	*F* = 1.64	0.197	0.023
		(10.29)	(7.22)	(2.92)				
Anxiety	F	13.21	4.93	4.00	Group	*F* = 37.68	< .001	0.35
		(8.72)	(5.94)	(4.37)	Gender	*F* = 1.30	0.26	0.009
	M	13.37	3.26	1.89	Group x Gender	*F =* 0.42	0.66	0.006
		(8.69)	(4.38)	(2.94)				
Stress	F	23.21	10.80	9.50	Group	*F* = 44.40	< .001	0.39
		(7.04)	(7.75)	(8.56)	Gender	*F* = 3.82	0.053	0.026
	M	21.05	9.05	5.47	Group x Gender	*F =* 0.27	0.76	0.004
		(12.44)	(5.67)	(3.94)				
PCL total	F	49.54	26.27	23.16	Group	*F* = 157.33	< .001	0.69
		(12.01)	(6.70)	(5.90)	Gender	*F* = 0.107	0.74	0.001
	M	50.95	26.63	20.00	Group x Gender	*F =* 0.98	0.38	0.014
		(11.78)	(6.71)	(2.52)				

^a^ Trauma-exposed controls

^b^ Non-trauma-exposed controls

Clinical data were assessed by separate 2 x 3 ANOVAs with sex (female, male) and group (PTSD, TC, NC) as between-subjects factors, which revealed a group main effect for each scale (see Table 1 for statistics). Sidak post-hoc analysis indicated that depression, anxiety and stress scales share a similar profile whereby the PTSD group scored significantly higher than both TC and NC groups (*p* < .001), but the difference between TC and NC groups were non-significant (*p* > .37). Sidak post-hoc analysis indicated that PCL total score was higher in the PTSD group than the TC group (*p* < .001, 95% CI [19.61, 27.98]), and higher in the TC group than the NC group (*p* = .015, 95% CI [0.74, 9.00]). There is a marginal sex main effect in stress (*p* = .053), but no significant sex main effect in these demographic scales. The group x sex interaction was non-significant for each scale (*p* > .05).

### Intentional recall

A mixed-design ANOVA with valence (negative, neutral, positive) as a within-subjects factor and sex (female, male) and group (PTSD, TC, NC) as between-subjects factors revealed effected reported in Table 2. There was a significant valence main effect such that participants recalled more negative images (*M* = 4.76, *SE* = 0.17) than both positive images (*M* = 2.43, *SE* = 0.14; *p* < .001, 95% CI [1.86, 2.80]) and neutral images (*M* = 1.82, *SE* = 0.13; *p* < .001, 95% CI [2.55, 3.33]); and more positive images than neutral images (*p* = .001, 95% CI [0.20, 1.02]). There was also a significant sex main effect, where images were recalled more by women (*M* = 3.43, *SE* = 0.13) than by men (*M* = 2.57, *SE* = 0.17). The group main effect was non-significant. The predicted interaction between sex, group and valence was also non-significant. All other effects were not significant.

**Table 2:** Statistics of effects on intentional recall and intrusive memory.

	Effects	*F*-Statistics	*P*	η_p_^2^	Observed Power
**Intentional recall**		* *	* *		
	Main (Valence)	*F*(2, 282) = 157.19	< .001	.527	1.000
	Main (Sex)	F(1, 141) = 16.20	< .001	.103	.979
	Main (Group)	*F*(2, 141) = 0.504	.766	.004	.132
	Sex x Valence	*F*(2, 282) = .03	.971	.000	.054
	Group x Valence	*F*(4, 282) = 1.02	.399	.014	.320
	Sex x Group	*F*(2, 141) = 0.27	.766	.004	.091
	Sex x Group x Valence	*F*(4, 282) = 1.32	.260	.018	.410
**Intrusive memory**		* *	* *		
	Main (Valence)	*F*(1.28, 180.55) = 37.46	< .001	.210	1.000
	Main (Sex)	*F*(1, 141) = 7.36	.007	.050	.769
	Main (Group)	*F*(2, 141) = 15.13	< .001	.177	.999
	Sex x Valence	*F*(1.28, 180.55) = 3.76	.044	.026	.550
	Group x Valence	*F*(2.56, 180.55) = 15.92	< .001	.184	1.000
	Sex x Group	*F*(2, 141) = 3.44	.035	.047	.638
	Sex x Group x Valence	*F*(2.56, 180.55) = 1.68	.180	.023	.399

### Intrusive memory

A mixed-design ANOVA with valence (negative, neutral, positive) as a within-subjects factor and sex (female, male) and group (PTSD, TC, NC) as between-subjects factors revealed effects reported in Table 2. The was a significant valence main effect such that participants reported more intrusions to negative images (*M* = 0.55, *SE* = 0.07) than both positive images (*M* = 0.09, *SE* = 0.03; *p* < .001, 95% CI [0.28, 0.64]) and neutral images (*M* = 0.04, *SE* = 0.02; *p* < .001, 95% CI [0.32, 0.70]); but there was no significant difference between the positive and neutral images (*p* = .32). The analysis revealed a sex main effect, where more intrusive images were reported by women (*M* = 0.31, *SE* = 0.04) than men (*M* = 0.14, *SE* = 0.05). This sex main effect was qualified by a significant interaction between sex and valence, such that more negative intrusive images were reported by women (*M* = 0.73, *SE* = 0.09) than men (*M* = 0.37, *SE* = 0.12; *p* = .016, 95% CI [0.07, 0.66]). The number of positive and neutral intrusive images reported by women and men were not significantly different.

There was also a group main effect, such that more intrusive images were reported by the PTSD group (*M* = 0.44, *SE* = 0.05) than the TC (*M* = 0.17, *SE* = 0.05; *p* = .001, 95% CI [0.10, 0.44]) and the NC (*M* = 0.06, *SE* = 0.05; *p* < .001, 95% CI [0.21, 0.56]) groups; and there was no significant difference between the TC and the NC groups (*p* = .300). This was qualified by interactions between group and valence, which revealed that more negative intrusions specifically were reported by the PTSD group (*M* = 1.15, *SE* = 0.13) compared to the TC group (*M* = 0.35, *SE* = 0.13; *p* < .001, 95% CI [0.39, 1.28]) and the NC group (*M* = 0.11, *SE* = 0.13; *p* < .001, 95% CI [0.64, 1.52]); and there was no significant difference between TC and NC groups (*p* = .454). The frequency of intrusions to the positive or neutral images did not differ among groups (*p* > .05).

**Fig 1:**
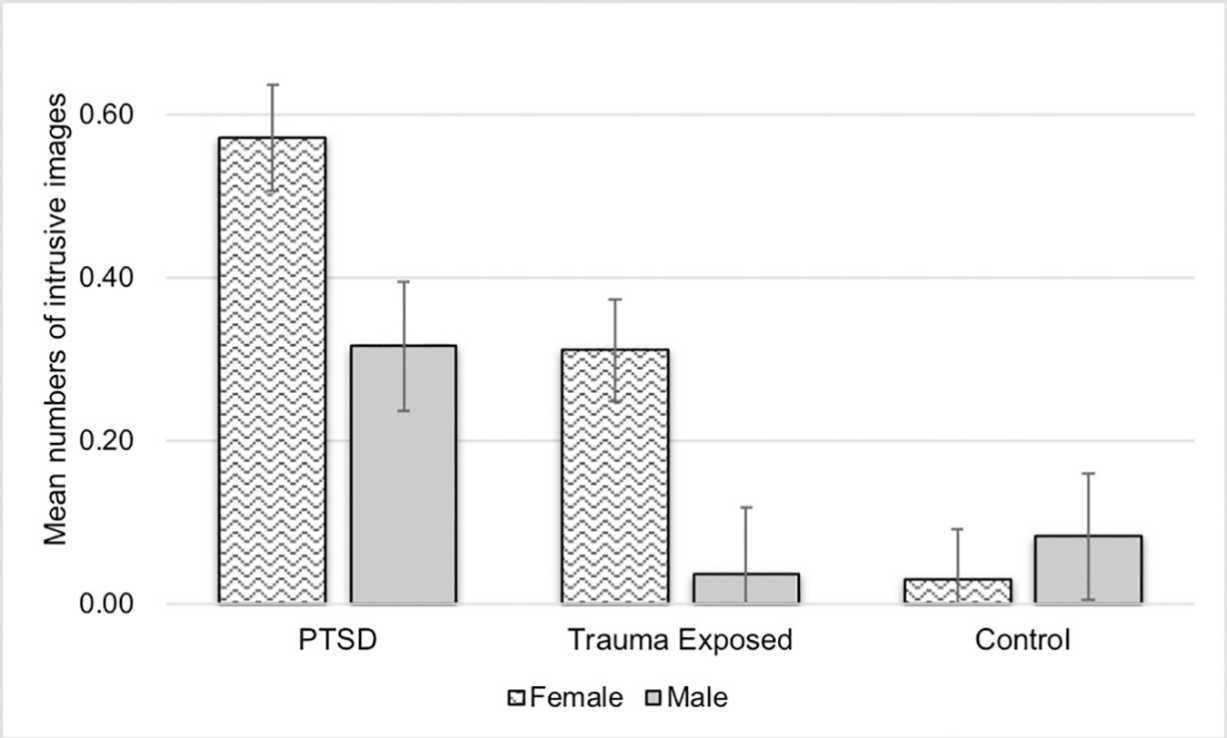
Mean numbers of intrusive images reported two days post-learning by males and females among the PTSD, trauma-exposed control (TC) and non-trauma-exposed control (NC) groups.

Importantly, there was a significant interaction effect between sex and group (Fig 1). Women reported more intrusive memories than men in the PTSD group (women: *M* = 0.57, *SE* = .07; men: *M* = 0.32, *SE* = 0.08; *p* = .014, 95% CI [0.05, 0.46]) and the TC group (women: *M* = 0.31, *SE* = .06; men: *M* = 0.04, *SE* = 0.08; *p* = .007, 95% CI [0.08, 0.48]), but there were no sex differences in the NC group (*p* = .572). The three-way interaction among sex, group and valence was not significant.

### Memory suppression

A Chi-square test of independence indicated a significant association between group and memory suppression, *X*^2^ (2, N = 137) = 27.78, *p* < .001. The PTSD group reported higher rates of memory suppression than both TE and NC groups. The association between sex and memory suppression was also significant, *X*^2^ (1, N = 137) = 5.23, *p* = .022, where women reported higher rates of memory suppression than men. The frequencies for suppression across groups and sex are displayed in Table 3. The analysis for individual-group subsamples revealed that more women reported memory suppression than men in the TC group, *X*^2^ (1, N = 47) = 4.31, *p* = .038, but such an association was not significant in the PTSD and the NC groups.

**Table 3:** Number of participants reporting attempts to suppress emotional images across gender and groups.

Group	Suppression		Total
	No	Yes	
Non-trauma-exposed controls			
Female	25	5	**30**
Male	15	1	**16**
Group total	**40**	**6**	**46**
Trauma-exposed controls			
Female	20	9	**29**
Male	17	1	**18**
Group total	**37**	**10**	**47**
PTSD			
Female	8	19	**27**
Male	9	8	**17**
Group total	**17**	**27**	**44**
All Groups			
Female	53	33	**86**
Male	41	10	**51**
Total	**94**	**43**	**137**

### Picture rating

The mixed design ANOVA revealed a significant valence main effect on subjective ratings of emotional valence, *F*(1.13, 58.82) = 209.50, *p* < .001, η_p_^2^ = .801. Negative images (M = 2.87, SE = 0.13) were rated lower than neutral images (M = 5.00, SE = 0.03; *p* < .001, 95% CI [1.81, 2.45]), and neutral images were rated lower than positive images (M = 6.43, SE = 0.13; *p* < .001, 95% CI [1.11, 1.76]). The mixed design ANOVA also revealed a significant valence main effect on subjective ratings of emotional arousal, *F*(1.90, 252.00) = 251.63, *p* < .001, η_p_^2^ = .654. Negative images (M = 5.57, SE = 0.18) were rated higher than positive images (M = 3.96, SE = 0.16; *p* < .001, 95% CI [1.24, 1.98]), and positive images were rated higher than neutral images (M = 1.77, SE = 0.10; *p* < .001, 95% CI [1.81, 2.56]).

## Discussion

This study examined whether there was greater negative memory consolidation in women with PTSD and/or with a history of trauma compared to men, and specifically examined if this was the case in two distinct forms of emotional memory: intentional recall (indexing strategic retrieval processes) and intrusive memory. Replicating previous research, negative images resulted in greater intentional recall and intrusive memories than neutral or positive images. Individuals with PTSD revealed significantly greater negative intrusive memories than the trauma-exposed or non-trauma controls, and women displayed significantly more intentional recall generally and reported significantly more intrusive memories of negative images than men (regardless of group). Key findings revealed sex differences in intrusive memories following trauma exposure as women with PTSD reported more intrusive images compared to men with PTSD, and this difference was not apparent in non-trauma-exposed individuals. Interestingly, not only did women with PTSD report more intrusions than men with PTSD, these sex differences were also apparent in the trauma-exposed control group. These findings support the hypothesis that women with PTSD will display significantly greater negative intrusive memories than men, but no significant sex x group effects were found for intentional recall.

The valence main effects for intentional recall and intrusive memories replicate many previous emotional memory studies which reveal that negative emotional images are associated with greater memory consolidation, leading to greater memory recall [33]. The interaction between group and valence for intrusive memories revealed that the PTSD group had significantly greater negative intrusive memories than both control groups, a finding that confirms previous research [15, 34]. This pattern is consistent with theoretical models highlighting that negative intrusive memories are key mechanisms involved in PTSD [12, 35].

There were sex main effects for intentional recall and intrusive memory revealing that women recalled more IAPS images and reported more intrusive memories than men. This main effect for intentional recall was found across valence, and a sex x valence interaction for intrusive memories showed that women had more intrusive memories specifically of negative images than men, but not positive or neutral images. These sex effects accord with an analogue emotional film study of healthy controls which found that women reported more intrusive memories than men [36]. This is also in line with the finding that women reported more involuntary memories than men when the task involved autobiographical memory [37, 38]. Given evidence that thought suppression increases the likelihood of intrusions [39, 40], it is possible that the greater suppression reported by women in the trauma-exposed group might contribute to the increased number of intrusive memories. This requires future research to specifically examine this question.

The key finding of the current study was the sex differences in intrusive memory observed in individuals with a history of trauma. The sex x group interaction revealed that women reported more intrusive memories than men, but only in those with PTSD or with a history of trauma, as this was not evident in the non-trauma controls. The fact that this gender effect was observed in both the trauma-exposed and PTSD groups is interesting to note, and suggests that trauma exposure per se may influence memory consolidation more in women than in men. Neuroimaging evidence also revealed that trauma exposure affects neural activity (in limbic regions involved in emotional memory) independent of PTSD symptoms [41]. Supporting evidence is found in a previous fMRI study in which women with PTSD or following trauma exposure were found to have significantly greater blood oxygen level in brainstem and midbrain arousal networks relative to men [42]. Therefore, trauma exposure may play a role in intrusion development.

A potential common mechanism thought to underlie intrusions which is also characteristic of both PTSD and trauma exposure, is arousal [14, 43, 44]. A key model of intrusions suggests that arousal during encoding is a key mechanism underlying intrusions [44] and arousal is also core to trauma exposure and a key characteristic of PTSD. Similar findings were revealed in healthy populations, as stress-induced cortisol during encoding was found to predict greater negative memory consolidation in healthy women [45]. It should be noted that this gender x group interaction only partially confirmed our hypotheses, as the increase of intrusive memories in women was not specific to negative images as predicted, but was a generalized effect across valence. Future studies should examine the impact of stress hormones to assess the potential role of arousal in intrusive memories.

Sex hormone is a potential influence on intrusive memories that were not examined in this study. Previous studies in women have found evidence that the luteal phase (characterised by high progesterone) and stress induction were associated with greater intentional recall [46] and intrusive memories [47, 48]. Similarly, another study found that intrusive memories of emotional films were reported more by women in the luteal phase than in the follicular phase, and those intrusions significantly correlated with progesterone [49]. In contrast, other studies found that estradiol levels influenced intrusive memories [50, 51], and one study found that estradiol-to-progesterone ratio predicted intrusions [48]. It is important to note that most of these studies recruited non-traumatized individuals. Although sex hormones may influence intrusive memories, the inconsistent finding highlight the need for future research to further delineate the relationship between specific hormones and intrusive memories. Given that both intrusive memories and estradiol are associated with limbic network activation [52, 53], and that estradiol modulates noradrenergic activity which is associated with intrusive memories [15, 54, 55], it is important to examine the potential interaction of sex and stress hormones on intrusive memories following trauma exposure.

In contrast to the intrusive memory data, the intentional recall measure did not reveal a significant group main effect or significant interaction with sex. Therefore, there was no evidence of greater recall of negative images in the PTSD group. This result is contrary to the common assumption that people with PTSD will recall more emotional than neutral stimuli, but the result is consistent with previous experimental memory studies [15, 56]. This may have resulted in part from the use of relatively generic emotional and negative stimuli (from the IAPS) which were not specifically tailored to each individual’s traumatic experience. The only significant effects for the intentional recall data were a valence main effect and a sex main effect. The sex main effect indicated that women recalled more images than men irrespective of valence, which is consistent with the previous finding that women had better semantic memory [57], included more accurate and detailed episodic memory and autobiographical memory [58, 59]. Similarly, the valence main effect revealed that negative and positive stimuli were better recalled than neutral images (with negative images recalled most of all), which is consistent with many previous emotional memory studies [36, 45, 60].

As noted previously, a limitation of this study is the lack of stress hormone and sex hormone data which should be included in future research studies. However, this study contributes preliminary findings of sex differences which provides a further rationale for examining sex hormone effects. A second limitation is that we examined sex differences (a biological distinction) without examining gender effects and the potential influence of gender and social roles. It is recognized there is increasing diversity in gender identification and there is a potentially powerful impact of social roles on emotional functioning, so future studies should include specific measures of gender and social roles. While it was speculated that women might recall more counts of traumatic experiences than men, it is a limitation that this study did not quantify the amount of trauma exposure. However, the conceptual and methodological issues in assessing trauma history highlight multidimensional challenges in obtaining reliable information [61], particularly when a person attempts to recall a full personal history. Further, to account for the significant age difference between the PTSD group and the non-trauma control group, the analyses of memory data were repeated with age as a covariate and revealed no change to the key factorial effects (see S1 File). Finally, the small number of intrusive memories in the data is challenging and may have led to floor effects in our data, however this is common in many intrusive memory studies. It is noted that this study measured the number of intrusive memories of the emotional stimuli presented to the participants, rather than intrusive memories of each individual’s personal trauma.

In conclusion, this study provides preliminary evidence of sex differences in intrusive memories of emotional images among individuals with a history of trauma. Specifically, women following trauma exposure, including those with PTSD, reported more intrusive memories of emotional images compared to trauma-exposed males and non-trauma controls. However, this effect was small, and it was not specific to negative stimuli. This preliminary finding highlights the need for future research to consider sex differences in memory, and to specifically investigate the impact of sex hormones and stress hormones on intrusive memories following trauma.

## Supporting information

S1 File A ZIP file containing the dataset and statistical analysis results reported in this manuscript. (ZIP)
